# A realistic bi-hemispheric model of the cerebellum uncovers the purpose of the abundant granule cells during motor control

**DOI:** 10.3389/fncir.2015.00018

**Published:** 2015-05-01

**Authors:** Ruben-Dario Pinzon-Morales, Yutaka Hirata

**Affiliations:** Neural Cybernetics Laboratory, Department of Computer Science, Chubu University Graduate School of EngineeringKasugai, Japan

**Keywords:** adaptive control, artificial cerebellum, robotics, firing rate model

## Abstract

The cerebellar granule cells (GCs) have been proposed to perform lossless, adaptive spatio-temporal coding of incoming sensory/motor information required by downstream cerebellar circuits to support motor learning, motor coordination, and cognition. Here we use a physio-anatomically inspired bi-hemispheric cerebellar neuronal network (biCNN) to selectively enable/disable the output of GCs and evaluate the behavioral and neural consequences during three different control scenarios. The control scenarios are a simple direct current motor (1 degree of freedom: DOF), an unstable two-wheel balancing robot (2 DOFs), and a simulation model of a quadcopter (6 DOFs). Results showed that adequate control was maintained with a relatively small number of GCs (< 200) in all the control scenarios. However, the minimum number of GCs required to successfully govern each control plant increased with their complexity (i.e., DOFs). It was also shown that increasing the number of GCs resulted in higher robustness against changes in the initialization parameters of the biCNN model (i.e., synaptic connections and synaptic weights). Therefore, we suggest that the abundant GCs in the cerebellar cortex provide the computational power during the large repertoire of motor activities and motor plants the cerebellum is involved with, and bring robustness against changes in the cerebellar microcircuit (e.g., neuronal connections).

## 1. Introduction

Cerebellar granule cells (GCs) are the smallest and most numerous neurons in the central nervous system of vertebrates (Ito, 2011). Four dendrites and a long axon that bifurcates in two parallel fibers characterize the GCs (Ito, 2011; Billings et al., 2014). Due to this specialized morphology, theoretical works, and computational studies have suggested that the GCs perform high dimensional lossless sparsification of incoming information, which is required at downstream cerebellar circuits to perform associative learning (Marr, 1969; Albus, 1971; Medina and Mauk, 2000; Schweighofer et al., 2001; DAngelo and Zeeuw, 2009), adaptive filtering (Fujita, 1982; Dean et al., 2010), binary addressing (Kanerva, 1988), and motor acquisition and consolidation (Galliano et al., 2013). Yet, reaching a consensus about the role of the abundant GCs has been hampered by outstanding challenges of isolating, recording, and stimulating these cells. Exploratory experiments with animal models have attempted to clarify the role of the GCs by abolishing completely or partially their output by blocking neurotransmitter release from all GCs (Wada et al., 2007; Kim et al., 2009), eliminating all GCs (De Zeeuw et al., 2004), or knocking down calcium channels from a majority of GCs (Galliano et al., 2013). Nonetheless, these works cannot address directly the role of the abundant GCs because they alter the balance at the input layer of the cerebellum and compensatory mechanisms might affect their conclusions. Thus, a different framework is required.

Understanding the functional consequence of the abundant GCs is not only important for deepening our knowledge of the biological system, but also for engineering applications that employ computational models of the cerebellum (Verschure and Mintz, 2001; Hofstotter et al., 2002; Carrillo et al., 2008; Tanaka et al., 2010; Garrido Alcazar et al., 2013; Yamazaki and Igarashi, 2013; Pinzon-Morales and Hirata, under review). Adequate selection of the number of GCs could improve the ratio of energy consumption and control performance, improve robustness, and flexibility of the cerebellar model (Newman, 2003). Yet, there has not been any evaluation in real world engineering applications. Thus, we tested the role of the GCs in a real world engineering application using our bi-hemispheric neuronal network model of the cerebellum (biCNN) that incorporates a realistic cerebellar network architecture and learning algorithm whose validity has been proved in both simulation and real-world experiments (Pinzon-Morales and Hirata, 2013, 2014a). The biCNN model enables us to isolate the GCs, knock down their output while maintaining the integrity of the cerebellar circuit, and evaluate the motor performance attained during control of different plants. Using this framework we can test the role of the numerous GCs from an engineering point of view.

We demonstrate that the abundant number of GCs is relevant for accomplishing adequate control performance across a diverse set of control plants and brings robustness to the biCNN model against changes in its initialization parameters (i.e., synaptic weights and synaptic connections). We also show that not all the GCs are required to govern each control plant. What is more, the minimum number of GCs required to maintain adequate control increases with the complexity of the control object (i.e., DOFs). Discussion about the relation between the number of GCs, motor performance attained, complexity of the control object, and robustness is presented.

## 2. Materials and methods

### 2.1. Overview of the bi-hemispheric neuronal network model of the cerebellum (the biCNN model)

**Figure 1:**
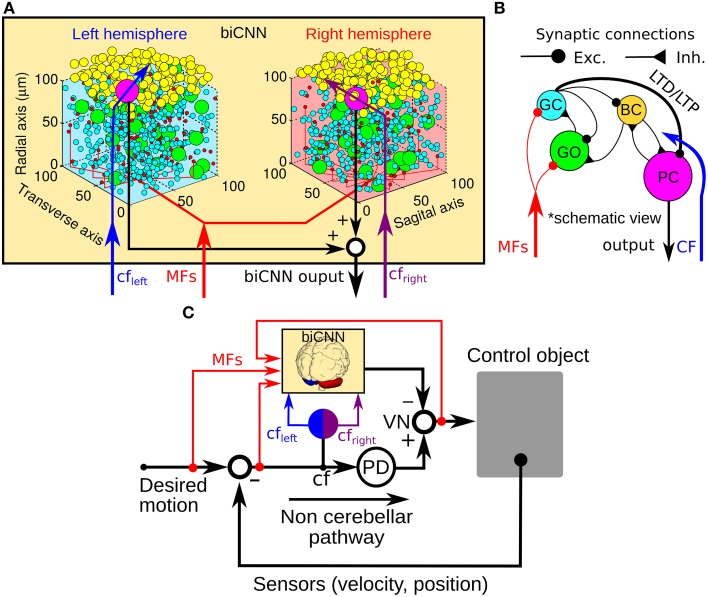
**General scheme of the biCNN model and its configuration for robot control. (A)** Right and left hemispheres of the biCNN model allocated in a 3D space. **(B)** Synaptic connections included in the biCNN model. **(C)** Wiring of the biCNN model during motor control. BC, basket/stellate cells; CF, climbing fiber; Exc., excitatory synapse; Inh., inhibitory synapse; GC, granule cell; GO, Golgi cell; MFs, mossy fibers; LTD, long-term depression; LTP, long-term potentiation; PC, Purkinje cell; PD, proportional and derivative controller; VN, vestibular nucleus.

Inspired by the neuronal circuit of the cerebellar cortex, we have previously developed a bi-hemispheric neuronal network model of the cerebellum (the biCNN model) (Pinzon-Morales and Hirata, 2013, 2014a) (Figure 1A). The biCNN model is freely available via repository (https://bitbucket.org/rdpinzonm/the-bicnn-model) or at the model database of the International Neuroinformatics Coordinating Facility (INCF) Japane Node, Cerebellar Platform (https://cerebellum.neuroinf.jp, id=1441). Briefly, the network contains the same neuron cell types and synaptic convergence/divergence ratios reported in the cerebellar cortex (Table 1, Figure 1B). Principally the biCNN model includes granule cells (GCs), Golgi cells (GOs), basket and stellate cells (BCs), and Purkinje cells (PCs) cells, cells whose physiological and anatomical properties have been well-characterized (Ito, 2011). Nonetheless, there are other less studied cerebellar cells that might have a role in the cerebellar algorithm such as Lugaro cells and unipolar brush cells (Dieudonné and Dumoulin, 2000). Synaptic connectivity includes excitatory projection from mossy fibers (MFs) to GCs and GOs, and from GCs to BCs and GOs via parallel fibers (PFs) of the GCs. Inhibitory feedback loop between GCs and GOs, and BCs and GOs, and mutual inhibitory loop between BCs and PCs (O'Donoghue et al., 1989; Dumoulin et al., 2001; Maex and Schutter, 2005; Pinzon-Morales and Hirata, under review) (Figure 1B). Mathematical models describing each neuron follow classical firing rate models (Pinzon-Morales and Hirata, 2013, under review), according to which the cell output is computed as the weighted summation of inputs passed through an activation function. For instance, the equation describing the firing rate of one PC is as follows:

(1) $$x_{\text{PC}}=y_{\text{PF}}W_{\text{PF-PC}}+y_{\text{BC}}W_{\text{BC-PC}}$$

(2) $$y_{\text{PC}}=\frac{1}{1+e^{-\sigma (x_{\text{PC}}-\mu )}}-0.5$$

where σ = 8, μ = 1/2, **x**_PC_ is the activity vector of all PCs before being processed by the sigmoid activation function **y**_PC_ (Equation 2) which produces the firing rates of PCs in the interval [0 1], **y**_PF_ and **y**_BC_ are the firing rates of GC and BC also in the interval [0 1], and **W**_PF−PC_ and **W**_BC−PC_ are the matrix of synaptic weights between PF-PC and BC-PC. The firing rates of the PCs from the left hemisphere are inverted (i.e., firing rate in the interval [-1 0]) and added to those from the right hemisphere to generate the output of the biCNN model in the interval [-1 1].

**Table 1:** **Convergence and divergence synaptic ratio of the biCNN model**.

	**Num. Cells**	**Divergence**	**Convergence**	
Mossy fibers (MF)^*^	562			
Golgi (GO)	56			
Granular (GC)	8192			
Basket/Stellate (BC)	548			
Purkinje (PC)	30			
MF→ GC		1:59	4:1	Solinas et al., 2010; Ito, 2011
MF→ GO		1:7	66:1	Solinas et al., 2010; Ito, 2011
PF→ GO		1:12	1639:1	Solinas et al., 2010; Ito, 2011
GO→ GC		1:586	4:1	Solinas et al., 2010; Ito, 2011
PF→ BC		1:3	41:1	Maex and Schutter, 1998; Ito, 2011
PF→ PC		1:4	1024:1	Ito, 2011
BC→ PC		1:7	110:1	Solinas et al., 2010
PC→ BC		1:55	3:1	Schilling et al., 2008
BC→ GO		1:3	28:1	Dieudonné and Dumoulin, 2000

* Number of MF inputs to the cerebellar model changes with the control plant.

The biCNN model included two networks with the same characteristics for the left and right hemispheres of the cerebellum. The construction of each network follows an 3D dimensional approach (Pinzon-Morales and Hirata, 2014a) according to which the first step is to place randomly each neuron inside a volume of the cerebellar tissue, here represented by a cube of edge length 100 μm (Figure 1B). Then each neuron is connected using a nearest-neighborhood rule and the convergence/divergence ratios of each cell type (Table 1). For instance, for a GC cell that receives 4 different MFs and 4 different GO inputs, the procedure connects the four closest MFs and GO cells. This procedure along with the random allocation of the neurons inside the cube, secure the singularity of each hemisphere while conserving the general characteristics of the cerebellar microcircuit. Random synaptic weights (**W**) are extracted from a normal distribution (μ = 0.9 and σ = 0.1 ∈ [0.8, 1]) and multiplied by a normalizing constant (*d*) that is cell dependent. *d* is determined as the inverse of the number of inputs of the same nature (excitatory or inhibitory) of each cell (Pinzon-Morales and Hirata, 2014a). A proportional and derivative (PD) controller, which is a feedback controller widely used in industry and other applications, is included in tandem with the biCNN model to provide the non-cerebellar and non-adaptive input to the vestibular nucleus (VN) that receives the firing rate of PC from left and right hemispheres and then produce the motor command (Figure 1C, PD).

Inputs to the biCNN model are carried by MFs and a climbing fiber (CF). MFs are postulated to provide desired motion signals, efference copy of motor commands, and sensory error signals (i.e., desired trajectory—actual trajectory) (Hirata and Highstein, 2001; Blazquez et al., 2003; Huang et al., 2013). The CF input on the other hand, has been proposed to carry an error signal that drives plasticity at the cerebellar cortex (Ito, 2013), specially at synapses between PFs and PCs. The current configuration of the biCNN model include long-term depression (LTD) and long-term potentiation (LTP) at PF-PC synapses (Ito, 2011) as described below:

(3) $$\Delta W_{{\text{PF}}_{i}-{\text{PC}}_{j}}(t)=\left\{ \begin{array}{l} -\gamma_{\text{LTD}}cf(t)y_{{\text{pf}}_{i}}(t)\text{ if }cf(t)>cf_{\text{spont}}\\ \;\gamma_{\text{LTP}}y_{{\text{pf}}_{i}}(t)\text{ otherwise} \end{array}\right.$$

where Δ*W*_PF_*i*_ − PC_*j*__(*t*) is the change in the synaptic weight between the *i*-th PF and the target *j*-th PC, *cf*(*t*) is the CF activity, *y*_pf_*i*__(*t*) is the firing rate of the *i*-th PF, and γ_LTD_ = 4 × 10^−6^ and γ_LTP_ = 0.3 × 10^−6^ are the learning rates for LTD and LTP, respectively. The threshold value *cf*_spont_ = 0.05 represents the spontaneous activity in CF that has been shown to encode non-preferred direction of sensory error (Hirata et al., 2006, 2007; Pinzon-Morales and Hirata, 2014a). Thus, each hemisphere is configured to receive a CF (Figure 1A, cf_right_ and cf_left_) carrying information from mainly one direction of the motion of the control object. For example in the case of the robot, the left hemisphere receives mainly forward sensory error (encoded in *cf*_left_(*t*) > *cf*_spont_) and small backward sensory error (encoded in *cf*_left_(*t*) < *cf*_spont_) (Pinzon-Morales and Hirata, 2014a). The opposite combination applies to the right hemisphere. The biCNN model is implemented in a Windows computer 4 × 3.33 Ghz Intel Core-i7 processor, memory: 16 GB running LabVIEW 2013 (see Pinzon-Morales and Hirata, 2014b for another implementation).

### 2.2. Control objects

**Figure 2:**
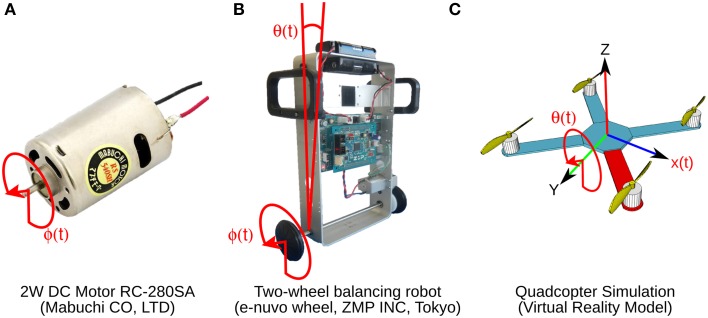
**Control objects and their control variables shown in red. (A)** Two watts direct current motor with a 1 DOF. Control variable is shaft angular position ϕ(*t*). **(B)** Two-wheel balancing robot with 2 DOFs. Control variables are wheel angular position ϕ(*t*) and body tilt angle θ(*t*). **(C)** Virtual model simulation of a quadcopter with 6 DOFs. Control variables are pitch θ(*t*) and horizontal position *x*(*t*).

Three control objects, a brushed DC motor, a two-wheel balancing robot, and a quadcopter are employed (Figure 2). The 2 W brushed DC electric motor (RC-280SA, Mabuchi CO, LTD, Japan. Figure 2A) generates a torque directly from DC current supplied. It is a control object with a single DOF. The motor's shaft is interfaced with an encoder circuit (ZMP INC., Japan) for providing angular position information [ϕ(*t*)], and a microcontroller board (e-nuvo CPU board, ZMP INC., Japan) in charge of communication with the implementation computer via USART Serial protocol. The MF inputs to the biCNN model for this control object are shown in Table 2. The PD controller for this plant is a position controller with *k*_*p*_ = 0.8 and *k*_*d*_ = 0.01 as PD constants, respectively. A virtual dynamical model simulation for this motor has been included in the repository of the biCNN model as an example (see Section 2.1 for the download links).

**Table 2:** **Mossy fibers for each control plant**.

**Object**	**Outputs (Sensors)**	**MFs**	**Scaling gain**
DC motor	ϕ(*t*): shaft ang. pos. (rad)	1- ref. shaft ang. pos.	0.1 rad^−1^
	$\dot{\phi }$(*t*): shaft ang. vel. (rad/s)	2- ref. shaft ang. vel.	0.19 rad/s^−1^
		3- shaft ang. pos. error	0.5 rad^−1^
		4- shaft ang. vel. error	0.07 rad/s^−1^
		5- efference copy	1 A^−1^
Balancing Robot	ϕ(*t*) wheel angle (rad)	1- ref. wheel ang. pos.	0.03 rad^−1^
	$\dot{\phi }$(*t*) wheel ang. vel. (rad/s)	2- ref. wheel ang. vel.	0.04 rad/s^−1^
	θ(*t*) body tilt ang. pos. (rad)	3- body tilt ang. pos. error	1 rad^−1^
	$\dot{\theta }$(*t*) body tilt ang. vel. (rad/s)	4- body tilt ang. vel. error	0.5 rad/s^−1^
		5- wheel ang. pos. error	0.1 rad^−1^
		6- wheel ang. vel. error	0.2 rad/s^−1^
		7- efference copy	0.5 A^−1^
Quadcopter	*x*(*t*) hor. pos. (m)	1- ref. hor. pos.	0.05 m^−1^
	θ(*t*) pitch (rad)	2- ref. pitch pos.	0.5 rad^−1^
	$\dot{x}$(*t*) hor. vel. (m/s)	3- hor. pos. error	0.05 m^−1^
	$\dot{\theta }$(*t*) pitch vel. (rad/s)	4- pitch error	0.6 rad^−1^
		5- hor. vel. error	0.05 m/s^−1^
		6- pitch vel. error	0.1 rad/s^−1^
		7- efference copy	0.3 A^−1^

Reference (ref.), angular (ang.), velocity (vel.), horizontal (hor.), and position (pos.).

Input for all control plants is in units of electric current (A).

MFs were repeated evenly to account for the 562 inputs in the biCNN.

The two-wheel balancing robot (e-nuvo wheel, ZMP INC, Tokyo. Figure 2B) is a 2 DOFs inverted pendulum system that is highly unstable and widely used in control engineering for testing control strategies (Li et al., 2013). It is equipped with a set of sensors including a motor encoder and a gyroscope, which provide wheel angle [ϕ(*t*)] and body tilt angle [θ(*t*)], respectively. The robot is also equipped with a USART chip to allow serial communication with the computer on which the biCNN model was implemented. The motion of the robot is driven by a single DC motor connected to its wheels. The MFs inputs carry the signals described in Table 2. Sampling frequency for the two-wheel balancing robot alike the DC motor is 10 ms. The PD controller in this control object is a parallel configuration of two controllers (body position controller: *k*_*p*_ = −18.017 and *k*_*d*_ = −2.511 and wheel position controller: *k*_*p*_ = −0.553 and *k*_*d*_ = −0.197) designed by following optimal settings for automatic controllers (Ziegler and Nichols, 1942; Li et al., 2013), so that the addition of both outputs (i.e., PD controller output) alone can stably operate the robot during a simple task (ϕ_*ref*_(*t*) = πsin(2 π 0.1 *t*), where ϕ_*ref*_(*t*) is the desired wheel angular position). A virtual dynamical model simulation for this balancing robot has also been included in the biCNN model repository as an example.

The quadcopter is a 6 DOFs system multirotor helicopter that is lifted and propelled by four brushless DC motors. The mathematical model describing its dynamics has been reported somewhere else (https://github.com/dch33/Quad-Sim). The MF inputs for this simulated quadcopter carry the signals described in Table 2. The four motors of the quadcopter are controlled by the action of four PD controllers corresponding to yaw, pitch, roll, and altitude, respectively. Controlling the displacement of the quadcopter in the X, Y, and Z plane is achieved by changing the reference point of pitch, roll and altitude controller, respectively. Here, we interface the biCNN model in the control loop for pitch [θ(*t*)]. Parameters of the controllers can be found in the virtual dynamical model simulation included in the biCNN model repository.

### 2.3. Experimental protocol

A control task was configured for each control object comprising at least 100 repetitions of the desired motion. In the case of the DC motor, the desired shaft position [ϕ_*ref*_(*t*)] is a sinusoidal motion at *f* = 0.5 Hz [i.e., ϕ_*ref*_(*t*) = πsin(2π 0.5 *t*)]. The balancing robot is commanded to follow a sinusoidal wheel motion [ϕ_*ref*_(*t*) = π sin(2 π 0.25*t*)] while the body tilt angle remains constant [90° with respect to the horizontal plane, θ_*ref*_(*t*) = 0], whereas the desired motion for the quadcopter is a sinusoidal horizontal (X-plane) motion with amplitude 2 m [i.e., *x*_*ref*_(*t*) = 2sin(2 π 0.2*t*)]. Amplitude and frequency of the desired motions were chosen to be between 80 and 90% of the maximum values that can be controlled for each plant in our setup. The number of active GCs in each hemisphere of the biCNN model was modified by knocking down the initial GC population (4000 GCs). Twelve numbers were considered i.e., 4, 10, 20, 40, 80, 200, 400, 800, 1000, 1600, 2000, or 4000 GCs. The numbers of GOs (27), MFs (257), BCs (267), and PC (1) in each hemisphere were kept constant. A scaling synaptic constant (1/number of knocked down GCs) was employed to compensate for the missing excitatory input to BCs, GOs, and PC. Since the attained motor performance might be affected by the initialization conditions of the biCNN model such as the random values of the synaptic weights and the random synaptic connections (see Section 2 for a description of the synaptic connections), five different sets of random synaptic weights and five tables of random synaptic connections are created to form a set of 25 permutations of initial conditions. Each control task was repeated 25 times for a given number of knocked down GCs (i.e., 25 × 12 = 300 experiments per control object). The yielded motor performance was measured cycle-by-cycle as the root mean square error (RSE) of the desired and yielded motion. Performance of the trained biCNN model was compared with that of the PD controller alone using One-Way ANOVA. Box plots are used to show statistical significance of the difference between the untrained (cycles #5–6) and trained (cycles #90–91, quadcopter and two-wheel balancing robot; cycles #180–181, DC motor) biCNN models. In these figures, the box represents the central 50% of the data. Its lower and upper boundary lines are at the 25 and 75% quantile of the data, and the central line shows the median of the data (*N* = 25 experiments × 2 cycles = 50 data points).

## 3. Results

We divided the experimental results into two parts with the purpose of studying the consequences of the number of GCs in the biCNN model during motor control. First, we show the behavioral consequences in terms of motor performance (see Section 2.3 for details about the experimental protocol), and second, we show the neural consequences at PC firing rates, PF-PC synaptic weights, and inputs to the GCs.

### 3.1. Behavioral consequences of the number of GCs

**Figure 3:**
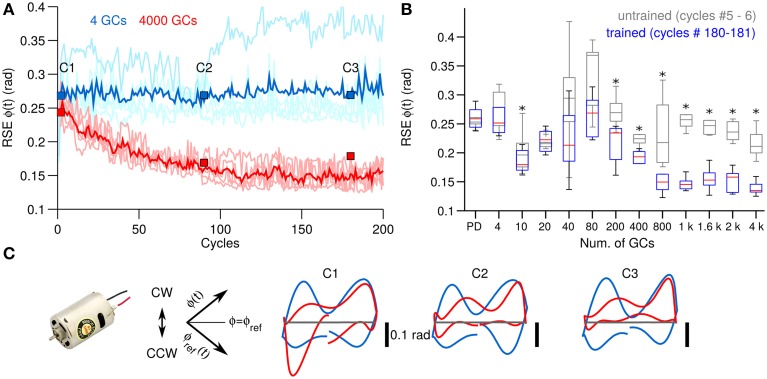
**Comparison of control performance with the number of GCs during control of the DC motor. (A)** Raw (light colors) and average (*N* = 25, bold colors) control performance in terms of RSE of ϕ(*t*) when using 4 GCs (blue lines) and 4000 GCs (red lines) per hemisphere in the biCNN model. Blue and red boxes labeled as C1, C2, and C3 show the cycles used in **(C)**. **(B)** Box plots showing the control performance (*N* = 50) vs. the number of GCs in the biCNN model at the beginning (cycles #5–6, gray boxes labeled as untrained) and the end (cycles #180–181, color boxes labeled as trained) of the experiments. Performance without the biCNN model (i.e., DC motor controlled only by the PD) is shown for reference. Asterisks show significant test (*p* < 0.05, One-Way ANOVA) between the PD and the trained biCNN model. **(C)** Yielded (Y-axis) vs. desired (X-axis) shaft angle position [ϕ(*t*)] (rotated −45°. Axes shown for reference).

Figure 3A shows the control performance of the DC motor (see Section 2.2 for detailed description of the control objects) in terms of the root mean square error (RSE) of the shaft angular position [ϕ(*t*)] with 4 and 4000 GCs (blue and red lines) in each hemisphere of the biCNN model as two examples of the GC size. ϕ(*t*) is depicted with the DC motor in Figure 2A. Average RSE of ϕ(*t*) (*N* = 25) is shown in bold blue and red lines. The RSE of ϕ(*t*) shows that the biCNN model with 4000 GCs adapted and improved the motor performance (RSE value was reduced on average 0.07 rad, 33.2% of the initial error value 0.20 rad), meaning that the PC learned the adequate motor commands to move the shaft of the DC motor to follow the desired motion. On the contrary, using 4 GCs produced highly variable performance and small improvement. Box plots in Figure 3B summarize the motor performance for the 12 sizes of GCs considered (see Section 2.3 for details about the experimental protocol) and for reference the performance when the biCNN model was disabled (i.e., DC motor controlled only by the PD) is shown as “PD.” Gray boxes were calculated at the beginning (cycles #5–6, labeled as “untrained”) and color boxes at the end (cycles #180–181, labeled as “trained”) of the experiment from the 25 different initialization conditions of the biCNN model. This result demonstrates that control performance of a DC motor with 1 DOF is improved by using the biCNN model in comparison with a PD controller alone (multiple comparisons, *p* < 0.05 for all sizes excepting 4, 20, 40, and 80 GCs). The best performance was produced with 1000 GCs (average RSE of ϕ(*t*) was reduced to 0.11 rad, 41.5% of the initial error value 0.25 rad). Using 40 or less GCs resulted in notably irregular performance and little improvement. Figure 3B also shows that increasing the number of GCs was accompanied by a reduction in the standard deviation of the RSE (e.g., with 80 and 1000 GCs the standard deviation of RSE was 0.14 rad and 0.041 rad, respectively) caused by changing the initial conditions. Therefore, increasing the number of GCs in the biCNN model during control of a single DOF system improves performance and reduces the variability due to changes in the initialization conditions.

Figure 3C shows a XY-plane constructed by plotting the yielded [ϕ(*t*)] against the desired [ϕ_*ref*_(*t*)] shaft position rotated by 45° (axis included for reference). Panels C1–C3, which correspond to the cycles shown in Figure 3A with equal labels, show the behavioral effects on the yielded shaft motion when 4 (blue lines) and 4000 (red lines) GCs were used. Four GCs resulted in trajectories that diverged from the ideal trajectory (Figure 3C gray lines) both in the positive (clockwise) and in the negative (counterclockwise) rotation of the shaft (shown in Figure 3C). On the contrary, the trajectories generated by using 4000 GCs progressively improved, especially in the clockwise direction.

**Figure 4:**
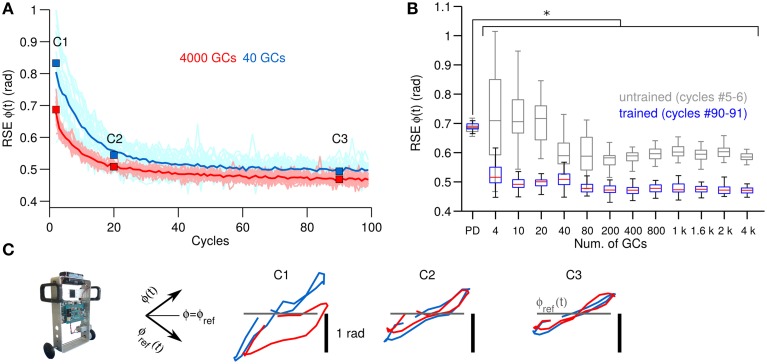
**Comparison of control performance with the number of GCs during control of the two-wheel balancing robot in the same format as Figure 3. (A)** Raw and average control performance in terms of RSE of ϕ(*t*) when using 40 GCs (blue lines) and 4000 GCs (red lines) per hemisphere in the biCNN model. **(B)** Box plots showing control performance (*N* = 50) vs. the number of GCs in the biCNN model at the beginning (cycles #5–6, gray boxes labeled as untrained) and the end (cycles #90–91, color boxes labeled as trained) of the experiments. Error bars show the standard deviation caused by the initialization conditions. Asterisks show significant test (*p* < 0.05, One-Way ANOVA) between the PD and the trained biCNN model. **(C)** Yielded (Y-axis) vs. desired (X-axis) wheel angle position [ϕ(*t*)] (rotated −45°. Axes shown for reference).

The next experiment consisted in verifying the behavioral consequences observed in a simple 1 DOF system with a more challenging control plant. For this purpose, the biCNN model is used for controlling a two-wheel balancing robot (Figure 2B), which is a system with 2 DOFs. Figure 4A, in the same format as Figure 3A, shows the control performance attained in terms of RSE of wheel angular position [ϕ(*t*)] with 40 and 4000 GCs (blue and red lines). In this control scenario, using 4 GCs and 10 GCs resulted in the robot falling in 15 out of 25 and 5 out of 25 repetitions, respectively. The control performances attained with 40 and 4000 GCs look alike except for the lower variability of the RSE of ϕ(*t*) achieved with 4000 GCs. This figure shows the adaptation capability of the biCNN model and the improvement in motor performance during control of a system with 2 DOFs. Similar to the previous control scenario, these results evidence that the PCs learned the adequate motor commands to move the two-wheel balancing robot to follow the desired motion.

Figure 4B, in the same format as Figure 3B, summarizes the motor performance for the 12 sizes of GCs considered. This figure shows the improvement in motor performance by using the biCNN model (untrained and trained performance, multiple comparisons, *p* < 0.05). In average the RSE was reduced by 0.5 rad or 45.5% of the initial error value of 1.1 rad. Little improvement in average motor performance was achieved beyond a certain number of GCs (80–200 GCs, Figure 4B color boxes). Nonetheless, increasing the number of GCs was accompanied by a reduction in the standard deviation of the RSE (error bars), especially during the early cycles. The biCNN model always outperformed the PD (shown as “PD” in Figure 4B in comparison with color boxes). Figure 4C in the same format as Figure 3C, presents the yielded wheel [ϕ(*t*)] motions when 40 (blue lines) and 4000 (red lines) GCs were used. Panel C1 shows that the wheel position trajectories during the beginning cycles were different for 40 and 4000 GCs. With 4000 GCs, the yielded wheel position presented relatively large deviation toward negative values, which corresponds to the backward motion of the two-wheel balancing robot. We have studied the asymmetry between forward and backward motion in the two-wheel balancing robot in our previous work (Pinzon-Morales and Hirata, 2014a) to produce asymmetrical adaptation in the biCNN model as in the real cerebellum. As the experiment progressed, the biCNN model evolved and the two-wheel balancing robot gradually approached to the ideal trajectory (gray line) regardless of the number of GCs (panel C3).

**Figure 5:**
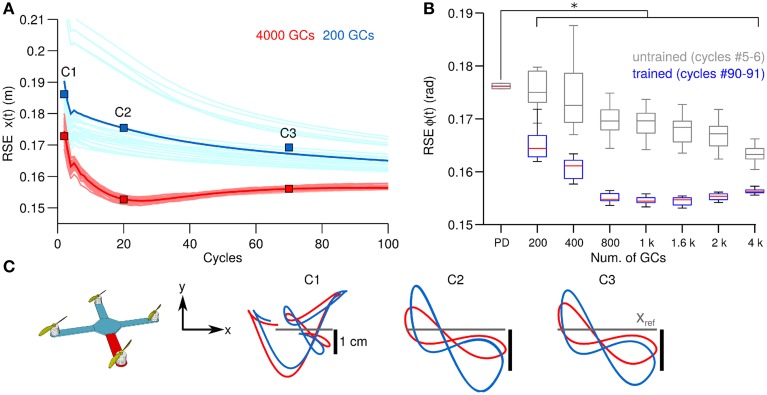
**Comparison of control performance with the number of GCs during control of the quadcopter in simulation in the same format as Figure 3. (A)** Raw and average control performance in terms of RSE of ϕ(*t*) when using 200 GCs (blue lines) and 4000 GCs (red lines) per hemisphere in the biCNN model. **(B)** Box plots showing control performance (*N* = 50) vs. the number of GCs in the biCNN model at the beginning (cycles #5–6, gray boxes labeled as untrained) and the end (cycles #90–91, color boxes labeled as trained) of the experiments. Error bars show the standard deviation caused by the initialization conditions. Asterisks show significant test (*p* < 0.05, One-Way ANOVA) between the PD and the trained biCNN model. **(C)** Hover plane (*z* = 3 *mt*) of the quadcopter showing desired (gray) and yielded motion (blue and red lines).

Thus, far we have shown the behavioral consequences of the number of GCs in the biCNN during control of a 1 DOF and a 2 DOFs real world systems. Next we evaluate the behavioral consequences in a simulated model with 6 DOFs (Figure 2C). Figure 5A, in the same format as Figure 3A, shows the control performance in terms of RSE of *x*(*t*) when 200 and 4000 GCs (blue and red lines) during control of the simulated quadcopter. In this control scenario, using less than 200 GCs resulted in convergence errors happening during the simulation. Similar to the control scenarios in real world, the biCNN model increased the control performance (average improvement of 0.02 m, 15% of the initial value 0.2 m) and reduced the variability [i.e., standard deviation of RSE of *x*(*t*)] in the yielded motion of the 6 DOFs system. The performance with the quadcopter simulation also shows that the biCNN model produced a RSE value at cycles around #20 lower than the converged value at the end of the experiment. This behavior was observed with more than 1000 GCs and only during simulation. In real world robot control the biCNN model consistently converged to the lowest RSE value attained during the experiments. This result suggests that noiseless inputs impact the learning in the biCNN model, whereas in real world robot experiments the noisy inputs aim the learning convergence, as reported in other feedforward neural networks (Jim et al., 1996). Figure 5B, in the same format as Figure 3B, summarizes the motor performance for the 12 sizes of GCs considered. The biCNN model with 200 GCs or more outperformed the control performance achieved by using only the PD controller (multiple comparisons, *p* < 0.05). Figure 5C shows the XY-plane in which the quadcopter hovers. The desired trajectory is shown in gray and the yielded motion with 200 (blue lines) and 4000 (red lines) GCs are shown. This result shows that increasing the number of GCs was accompanied by reduced displacement in the y-axis (panel C1 in comparison with panel C3) away from the ideal trajectory (Figure 5C gray lines). Therefore, in this simulated system with 6 DOFs larger numbers of GCs in the biCNN model increase the control performance and reduce variability caused by the initial conditions as in the real world control objects tested above.

**Figure 6:**
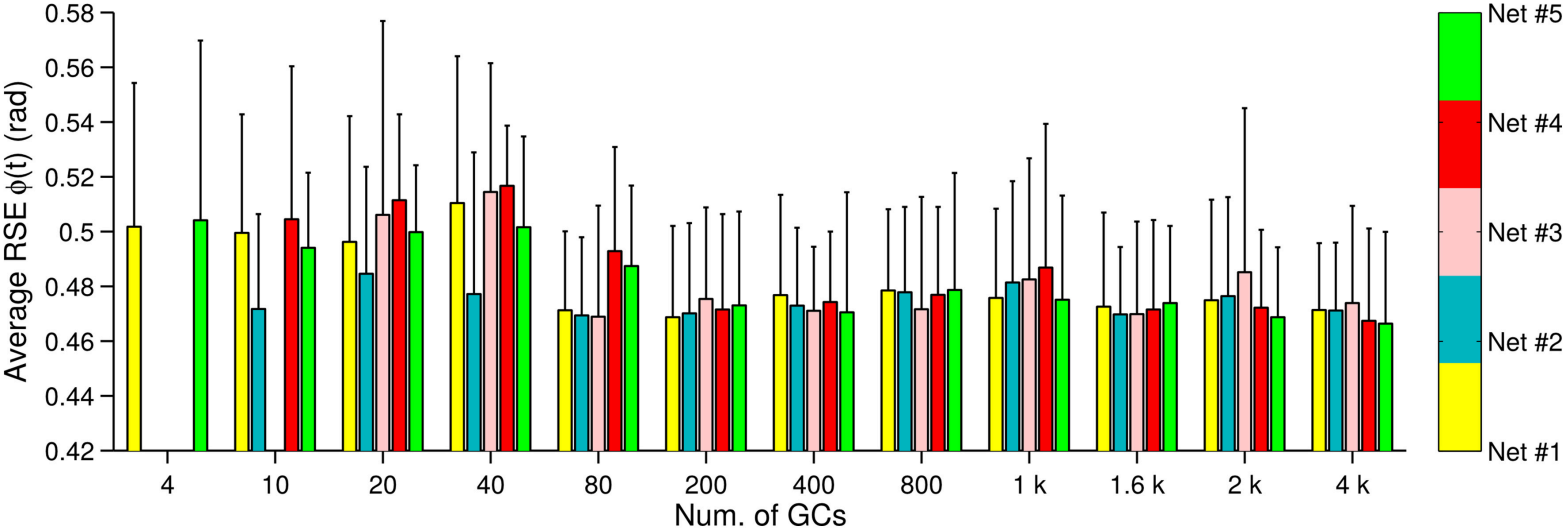
**Control performance and variability due to the initial conditions**. Bars represent a unique synaptic connections table labeled as Net #1–#5, whereas the error bars show the standard deviation due to the synaptic weights (*N* = 5).

We have shown in Figures 2B, 3B, 4B that the motor performance is affected by the number of GCs and also by the initial conditions. The initial conditions caused large variations in the control performance when using small numbers of GCs (<1000 GCs). There are two initial conditions in the biCNN model responsible for this variation, namely, synaptic weights and synaptic connections. Here, we evaluate the contribution of each initial condition to the overall variability in motor performance during control of the two-wheel balancing robot (variability with the DC motor and the quadcopter showed similar results). The 300 experiments were separated into 5 groups (referred to as Net #1–5) of 60 experiments each. The 60 experiments belonging to a given group share the same synaptic connections but differ in the number of GCs and set of random initial synaptic weights (12 × 5 sets of synaptic weights = 60, 60 × 5 Nets = 300 experiments). Figure 6 shows the average (bars) and standard deviation (error bars) of the RSE of ϕ(*t*) for each Net and number of GCs. This figure shows that three (Net #2, 3, and 4) out of five groups with 4 GCs failed to control the two-wheel balancing robot. Similarly, with 10 GCs Net #3 failed to control the robot. Figure 6 shows that increasing the number of GCs reduced the variability caused by changing the synaptic connections (average RSE, i.e., difference between bars height) and to a lesser degree the variability caused by the initial synaptic weights (standard deviation of RSE: error bars). Therefore, the synaptic connections produced the major part of the variability of the control performance with small numbers of GCs (<800). When the biCNN model included more than 1000 GCs disregarding of the control object, the variability due to both initial conditions was compensated.

### 3.2. Neural consequences of the number of GCs

**Figure 7:**
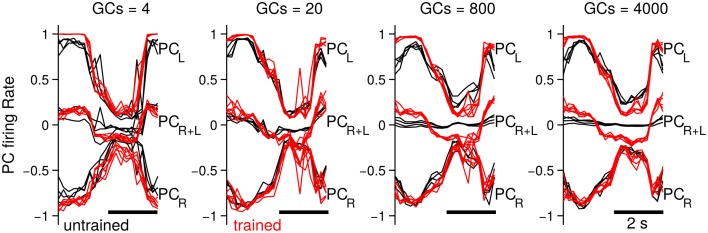
**Firing rate of the PCs in each hemisphere (PC_L_ and PC_R_) and their sum (i.e., input to the VN, labeled as PC_R+L_) when the biCNN model contained 4, 20, 800 and 4000 CGs cells per hemisphere**. Firing rate at the beginning (black traces, cycles #5–10) and end (red traces, cycles #90–95) of the experiment is shown labeled as untraining and trained, respectively.

Up to now we have investigated the behavioral consequences of the number of GCs in the biCNN model. In this section we evaluate the neural consequences, namely, PC firing rates, PF-PC synaptic weights, and the MF and GO inputs to GCs. The particular results for the two-wheel balancing robot are shown here. Results with the DC motor and quadcopter are presented in Supplementary Figures 1, 2, which followed similar trends to those of the two-wheel balancing robot. First, we studied the effects in the firing rate of the PCs. Figure 7 shows the firing rate of the PCs in the left (PC_L_) and right (PC_R_) hemisphere, and their sum (PC_R+L_), which corresponds to the cerebellar input to the VN with 4, 20, 800, and 4000 GCs. For the sake of comparison this figure presents five cycles aligned and superimposed at the beginning (cycles #5–10) and the end (cycles #90–95) of the experiment. Comparison of the firing rates evidences a change in the cerebellar input to the VN (i.e., PC_R+L_) caused by the number of GCs. When fewer (<80) GCs were used, the default firing rates of PC_L_ and PC_R_ at the beginning of the experiment did not cancel each other (Figure 7, 4 and 20 GCs black lines labeled as PC_R+L_), contrary to the case when more than 200 GCs were used. This means that the cerebellar input to the VN was carrying a default modulation in its firing rate with less than 200 GCs. Such a modulation was not learned and probably unrelated to the control task. On the contrary, with more than 200 GCs, PC_R+L_ did not present any modulation, meaning that the cerebellar input to the VN was neutral to the control task. The default information in PC_R+L_ (Figure 7 red traces) endured to the last cycles of the experiments. With 4 or 20 GCs the evolved PC_R+L_ presented similar shape as the firing rate in the early training (i.e., a bias information). With 800 or 4000 the evolved PC_R+L_ corresponded to the motor command required during the control of the two-wheel balancing robot (Supplementary Figure 1 shows similar results with the DC motor and the quadcopter).

**Figure 8:**
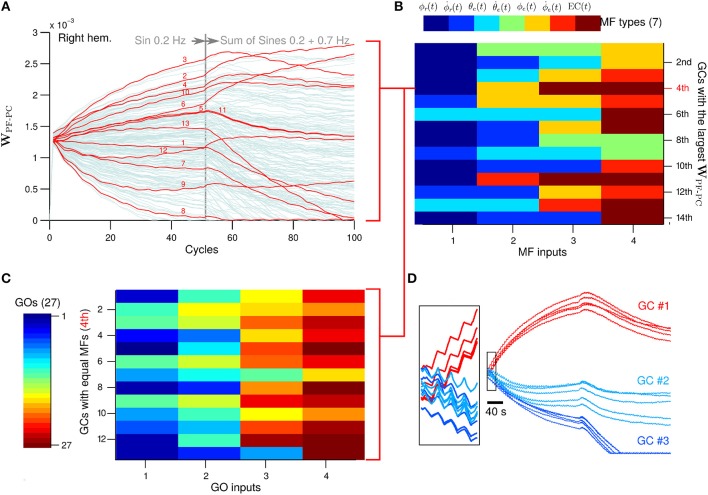
**(A)** GCs that share the same types of MFs inputs of the GC that achieved the 4th largest **W**_PC-PF_. Numbers in red corresponds to the GCs number in the y-axis in **(C)**. **(B)** The four MFs inputs of the 14th GCs with largest **W**_PC-PF_. The color indicates a different type of MF. **(C)** GO inputs of those GCs that have the same MFs inputs as the 4th GCs with largest **W**_PC-PF_. **(D)** Behavior of the **W**_PC-PF_ of three representative GCs when the random initial weight was changed and the connectivity table remained the same (*N* = 5).

Next we evaluate the PF-PC synapses, which are the sole plastic loci in the current configuration of the biCNN model. To observe additional neural changes, the desired wheel motion (see Section 2.3 for more details about the experimental protocol) was changed at cycle #50 to a more difficult motion corresponding to a sum of sines (ϕ_*ref*_(*t*) = π sin(2 π 0.2*t*) + sin(2 π 0.7*t*), θ_*ref*_(*t*) = 0) and left for 50 more cycles. The sum of sines is a much more difficult motion for the robot. In fact, disabling the biCNN model output (i.e., robot controlled only by the PD) completely abolished the control of the robot. We analyzed the synaptic weights between GCs and PC (**W**_PF−PC_) in search of extra insights into the role of the GCs (Figure 8A with 1000 GCs in the right hemisphere; other GCs and left hemisphere presented similar behavior). During the first 50 cycles corresponding to the single sinusoidal task, the PC-PF synapses diverged from the initial value by action of LTD (48.6% of GCs) and LTP (51.4% of GCs). Those GCs that carried relevant information to reduce the error signal in the CF input had their synaptic weight presumably increased, whereas those not relevant to reduce the error had their weight unchanged or decreased. Interestingly, at cycle #50 when the desired motion was changed, some GCs that had their synaptic weight decreased started to be potentiated (10.3%) and others further depressed (in total 75.1%). Similarly, some of the GCs that presented large synaptic weights had their weights decreased (34.0%) or more potentiated (in total 24.9%) (Supplementary Figure 2 shows similar results with different number of GCs). To discard the effect of the random initial synaptic weight in this result, Figure 8D shows the five different initial synaptic weights belonging to three representative GCs sharing the same synaptic connectivity (i.e., same Net). This figure demonstrates that despite the random initial values the global behavior of the synaptic weights follows a similar trend.

Next we investigated the MF and GO inputs to the GCs that had their **W**_PF−PC_ synaptic weight preferably potentiated (referred to here as “best GCs”) during control of the two-wheel balancing robot because those GCs are presumably best suitable for the control task. Once the best GCs were identified based on their evolved **W**_PF−PC_ synaptic weight, we analyzed their patterns of MF inputs and asked if those patterns are exclusively presented in the best GCs. Such a relationship would indicate that a particular pattern of MF inputs is well-suited for the control task. The GCs were sorted from largest to smallest **W**_PF−PC_ synaptic value at cycle #50. Each GC makes synapses with the four closest MFs in the 3D cube of cerebellar cortex in the biCNN model (see Section 2 for details about the network construction), and thus, each GC has a random combination of MFs inputs. Figure 8B shows the four MF inputs of the 14 best GCs (i.e., 14 largest **W**_PF−PC_) found, and the seven different types of MFs derived from the two-wheel balancing robot (see Section 2.2 for a description of the MFs for the current setup) coded by colors. The color matrix suggests that each of the 14 best GCs have a unique pattern of MF inputs. For instance, the GC with the 4th largest **W**_PF−PC_ receives MF inputs carrying desired wheel angle [dark blue, ϕ_*r*_(*t*)], wheel angle error [yellow, ϕ_*e*_(*t*)], and two MFs carrying efference copy [dark red, *EC*(*t*)]. However, this pattern of MF inputs is not unique to the 4th best GC. Figure 8A shows in red the **W**_PF−PC_ of all the GCs (13 out of 1000) that share the same pattern of MF inputs as the 4th best GC. It can be observed that some GCs were depressed and others were potentiated during the initial 50 cycles of the experiment. Therefore, the MFs inputs are not the only discriminant characteristic of those GCs preferably potentiated. The only option remaining to determine the convergent value of **W**_PF−PC_ is the GO inputs to the GCs. Each GC receives four GO inputs connected in the same fashion as the MF inputs (i.e., the fourth closest GOs). Figure 8C shows the four GO inputs (out of 27 available) for each of the GCs that share the same MF inputs as the best 4th GC. The order of the GCs follows the numeration shown in red in Figure 8A. This figure shows that each of the 13 GCs sharing the same MFs has different GO inputs, and therefore, each GC is processing a unique combination of inputs that yielded the different **W**_PF−PC_ values based upon the relevance of each GCs to the control task.

## 4. Discussion

We have investigated the role of the abundant GCs in a bi-hemispheric neural network (biCNN) model of the cerebellum compatible with cerebellar cortex anatomy and physiology during both real-world and simulated engineering applications. The biCNN model allows us to knock down the output of the GCs, preserving the integrity of the cerebellar microcircuit. It also allows us to observe the behavioral and neural consequences during control of different control objects differing in their degrees of freedom (DOFs). We considered a direct current motor with 1 DOF, an unstable two-wheel balancing robot with 2 DOFs, and a simulated model of a quadcopter with 6 DOFs. In this context the biCNN model presented a convenient framework to assess the role of the abundant GCs. We showed that all the control objects can be successfully controlled with a small number of GCs that depends upon the complexity of the control object. Further increase of the number of active GCs reduces the variability of control performance due to changes in the initialization parameters of the biCNN model. Hence, we suggest that the abundant GCs in the cerebellar cortex bring robustness against changes in the cerebellar microcircuit (e.g., neuronal connections), and as previously suggested, they provide the storage and computational power required for the PCs during the large repertoire of motor commands and motor plants the cerebellum is involved with. Below we discuss the essential role of GCs and we compare the biCNN model with other models of the cerebellum.

### 4.1. Essential role of the GCs

GCs are small, densely packed, and have a unique morphology with four dendrites and an axon that bifurcates in two parallel fibers. This is a set of remarkable features that suggest GCs have a high input sensitivity required for processing incoming information (Marr, 1969; Albus, 1971; Medina and Mauk, 2000; DAngelo and Zeeuw, 2009; Billings et al., 2014). There has been a popular theory proposing that GCs transform the incoming information into a higher dimensional, sparse representation (Marr, 1969; Porrill and Dean, 2007; Ito, 2011), which allows the downstream cerebellar circuits to perform associative learning (Marr, 1969; Albus, 1971; Medina and Mauk, 2000; Schweighofer et al., 2001; DAngelo and Zeeuw, 2009), adaptive filtering (Fujita, 1982; Dean et al., 2010), and binary addressing (Kanerva, 1988). Furthermore, the limited number of input synaptic connections (i.e., four dendrites) allows optimal lossless space encoding (Billings et al., 2014). A consequence that follows is that not all the GCs are required for a particular motor task, since only a fraction of the population is active at any time (i.e., sparse representation). Computational and experimental evidence have confirmed this premise. Schweighofer's model that implements unsupervised learning of GCs sparse coding showed that basic motor performance can be normal despite a small number of GCs (Schweighofer et al., 2001). Likewise, behavioral experiments with mutant mice with impaired GC output showed that motor performance during different motor tasks was intact (Galliano et al., 2013). In line with this evidence, the results of our behavioral experiments with both real world and simulated control objects showed that only a fraction of the GCs population is required for performing successfully a specific control task (DC motor, 10 GCs Figure 3A; two-wheel balancing robot, 40 GCs Figure 4A; quadcopter, 200 GCs Figure 5A). Our experiments further showed that increasing the number of GCs reduces the variability in the results caused by changing the initialization synaptic weights and the synaptic connections (Figure 6). This suggests that the abundant number of GCs brings robustness to the cerebellar circuit in two-ways; first, a large number of GCs mean that a vast repertoire of input patterns and output control objects can be adequately coordinated, and second, in the case of a structural reconfiguration (e.g., injuries, aging) the integrity of the cerebellar circuit and its functions can be preserved. From an engineering point of view, using a large number of GCs brought flexibility and robustness to the biCNN model as a controller because different types of control objects could be controlled. For instance, given a biCNN model with 200 GCs, a quadcopter, or a two-wheel balancing robot, or a DC motor could be adequately controlled. However, if the control task is fixed, the number of GCs can be reduced to find a compromise between the control performance and the energy/hardware requirements of the biCNN model. We hypothesize that increasing the number of DOFs of the control object would be accompanied by an increase of the minimum number of GCs needed to adequately control the object. Also, if the complexity of the control task is increased then the number of GCs that need to be recruited to produce the adequate motor command is bound to increase. To test this hypothesis and draw the exact relationship, systematic evaluation on the performance of the biCNN model with a variate set of control objects is required in a future study.

Our results are also in line with the prediction that the loop formed by the feedback and feedforward pathways between GC–GO, and MFs–GO–GC, respectively, support the sparsification of the incoming information at the GCs (Porrill and Dean, 2007). We found that the pattern of MF inputs of the GCs preferably potentiated during the control tasks did not provide enough features to discriminate the GCs. However, when we included in the classification the patterns of GO inputs to those GCs it was possible to identify unequivocally each GC. Therefore, the role carried out by the GCs is accentuated by the inhibition from GO in our biCNN model as in the real cerebellum.

### 4.2. Comparison with other models

Computational models of the cerebellum and their successful application in engineering have extensively been reported. Moreover, applications in robotic setups are also prominent and include control of the eye plant (Kettner et al., 1997), control of pneumatic muscles (Lenz et al., 2009), control of robotic arms (Kawato and Gomi, 1992; Eskiizmirliler et al., 2002; Garrido Alcazar et al., 2013), control of mobile robots (Verschure and Mintz, 2001; Hofstotter et al., 2002), and control of inverted pendulum systems (Ruan and Chen, 2011; Pinzon-Morales and Hirata, under review). The biCNN model has been employed previously to reproduce asymmetrical motor learning (Pinzon-Morales and Hirata, 2014a). The biCNN model is compatible with other models regarding the anatomical description of the cerebellar cortex (Solinas et al., 2010), employs a 3D approach for construction of the network connections following biological densities of neurons (Solinas et al., 2010), and includes a biologically plausible learning rule (Ito, 1998). We have also shown that the biCNN model is suitable for implementations using real-time, stand alone devices (Pinzon-Morales and Hirata, 2014b). In contrast to spiking neuron models of the cerebellum (Hofstotter et al., 2002; Garrido Alcazar et al., 2013; Yamazaki and Igarashi, 2013), due to the level of abstraction in our biCNN model (i.e., firing rate neuron models), spike patterns and temporal or spatial effects were not possible to evaluate. This would require the construction of a cerebellar network with spiking neuronal models that could endanger the real-time real-world application in control engineering. Finally, the biCNN model includes plasticity at synapses between GCs and PCs. However, other sites of plasticity in the cerebellum and their involvement in motor learning have been argued (McElvain et al., 2010; Gao et al., 2012; Garrido Alcazar et al., 2013), such as the synapses between molecular layer interneurons and PCs. Including other sites of plasticity remains in a future improvement of the biCNN model. The biCNN model is freely available via repository (https://bitbucket.org/rdpinzonm/the-bicnn-model) or at the model database of the International Neuroinformatics Coordinating Facility (INCF) Japane Node, Cerebellar Platform (http://cerebellum.neuroinf.jp, id=1441).

### 4.3. Experiment suggested by the model

Our results suggest an interesting prediction that could be tested experimentally: GOs accentuate the transformation of incoming information from MFs at GCs. Testing this hypothesis would require precise control over the output of the GOs, so that GO output can be knocked down while preserving the integrity of the other cerebellar cells. Manipulation of ion channel expression in GO membranes as shown in GCs (Galliano et al., 2013) is an interesting approach to follow. Once the output of GOs is under control, a set of simple and complex motor tasks such as gain and phase modulation of the vistibuloocular reflex can be assembled to assess the effects of reduced GO numbers on motor performance and motor memory consolidation. Results with our model predict that the motor impairment would be higher than that produced by knocking down the output of GCs.

### Conflict of interest statement

The authors declare that the research was conducted in the absence of any commercial or financial relationships that could be construed as a potential conflict of interest.

## Supplementary material

Supplementary Figure S1 **(A)** Firing rate of the PCs in each hemisphere (PC_L_ and PC_R_) and their sum (i.e., input to the VN, labeled as PC_R+L_) when the biCNN model contained 4, 40, 1000, and 4000 CGs cells per hemisphere during control of the DC motor. Firing rate at the beginning (black traces) and end (red traces) of the experiment is shown labeled as Early training and Trained, respectively. **(B)** Firing rate of the cerebellar input to the VN, labeled as PC_R+L_ when the biCNN model contained 200, 800, 1600, and 4000 CGs cells per hemisphere during control of the quadcopter.

Supplementary Figure S2 **Synaptic weights **W**_PC−PF_ during control of the two-wheel balancing robot**. Red lines highlight some weights as example. **(A)** 200 GCs. **(B)** 4000 GCs.
