# Supplementary figures and images for: A realistic bi-hemispheric model of the cerebellum uncovers the purpose of the abundant granule cells during motor control
Source: Front Neural Circuits. 2015 May 1;9:18. doi: 10.3389/fncir.2015.00018 (PMC4416449; doi:10.3389/fncir.2015.00018)

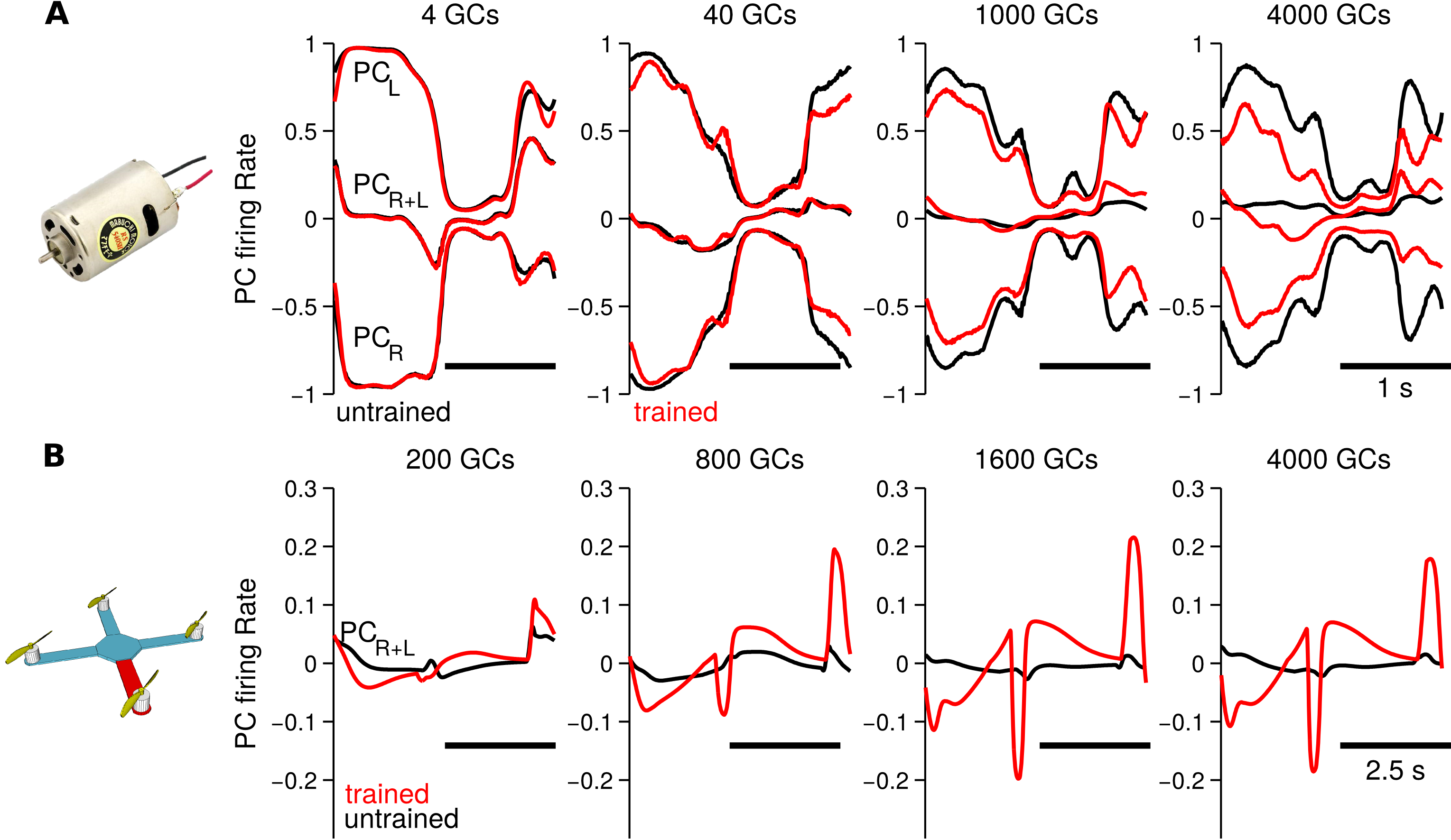

Supplement: Supplementary Figure S1 — (A) Firing rate of the PCs in each hemisphere (PCL and PCR) and their sum (i.e., input to the VN, labeled as PCR+L) when the biCNN model contained 4, 40, 1000, and 4000 CGs cells per hemisphere during control of the DC motor. Firing rate at the beginning (black traces) and end (red traces) of the experiment is shown labeled as Early training and Trained, respectively. (B) Firing rate of the cerebellar input to the VN, labeled as PCR+L when the biCNN model contained 200, 800, 1600, and 4000 CGs cells per hemisphere during control of the quadcopter. [file Image1.TIFF]

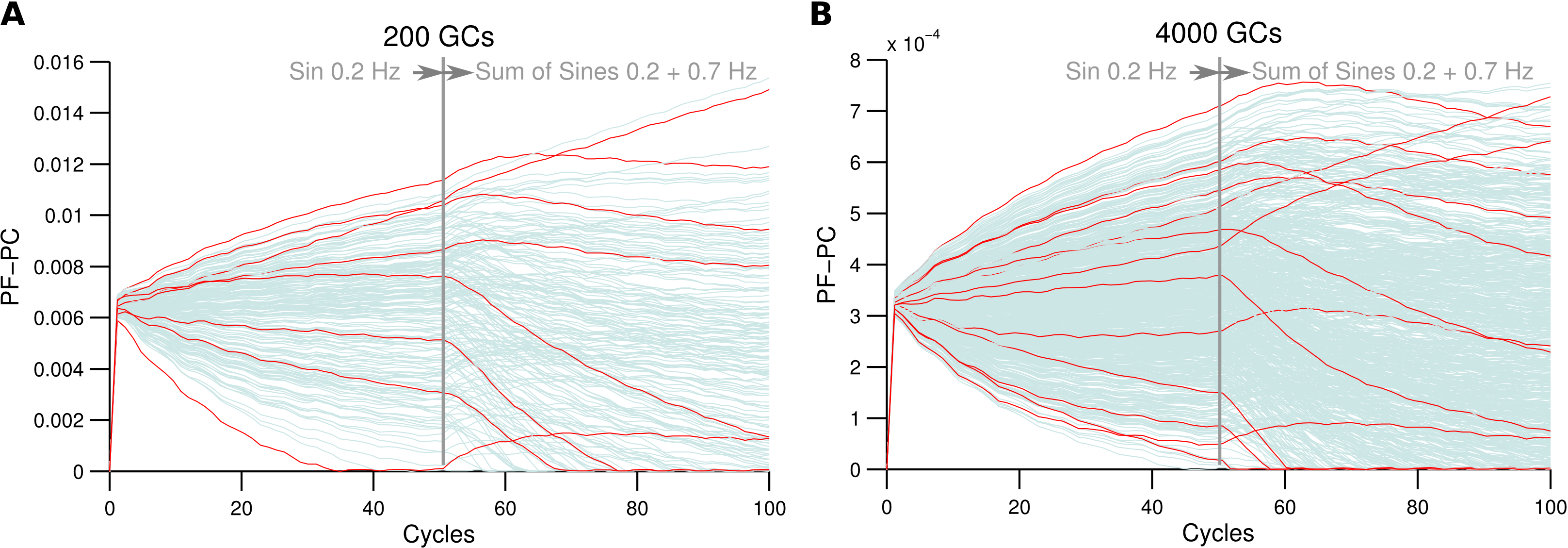

Supplement: Supplementary Figure S2 — Synaptic weights WPC−PF during control of the two-wheel balancing robot. Red lines highlight some weights as example. (A) 200 GCs. (B) 4000 GCs. [file Image2.TIFF]
